# Small non-coding RNA signature in multiple sclerosis patients after treatment with interferon-β

**DOI:** 10.1186/1755-8794-7-26

**Published:** 2014-05-17

**Authors:** Bruna De Felice, Paolo Mondola, Anna Sasso, Giuseppe Orefice, Vincenzo Bresciamorra, Giovanni Vacca, Elio Biffali, Marco Borra, Raimondo Pannone

**Affiliations:** 1 Department of Life Sciences, University of Naples II, Via Vivaldi 43, Caserta 81100, Italy; 2 Department of Clinical Medicine and Surgery, University Federico II of Naples, Corso Umberto I, Naples 80131, Italy; 3 Zoological Station Anton Dohrn, Villa Comunale, Piazza Vittoria, Napoli 80121, Italy; 4 DISTABIF, Department of of Environmental, Biological and Pharmaceutical Sciences and Technologies, University of Naples II, Via Vivaldi 43, 81100 Caserta, Italy

**Keywords:** Multiple sclerosis, IFN-β, sncRNA, microRNA, snorRNAs

## Abstract

**Background:**

Non-coding small RNA molecules play pivotal roles in cellular and developmental processes by regulating gene expression at the post-transcriptional level. In human diseases, the roles of the non-coding small RNAs in specific degradation or translational suppression of the targeted mRNAs suggest a potential therapeutic approach of post-transcriptional gene silencing that targets the underlying disease etiology. The involvement of non-coding small RNAs in the pathogenesis of neurodegenerative diseases such as Alzheimer’s , Parkinson’s disease and Multiple Sclerosis has been demonstrated. Multiple sclerosis (MS) is an autoimmune disease of the central nervous system, characterized by chronic inflammation, demyelination and scarring as well as a broad spectrum of signs and symptoms. The current standard treatment for SM is interferon ß (IFNß) that is less than ideal due to side effects. In this study we administered the standard IFN-ß treatment to Relapsing-Remitting MS patients, all responder to the therapy; then examined their sncRNA expression profiles in order to identify the ncRNAs that were associated with MS patients’ response to IFNß.

**Methods:**

40 IFNß treated Relapsing-Remitting MS patients were enrolled. We analyzed the composition of the entire small transcriptome by a small RNA cloning method, using peripheral blood from Relapsing-Remitting MS patients at baseline and 3 and 6 months after the start of IFNß therapy. Real-time qPCR from the same patients group and from 20 additional patients was performed to profile miRNAs expression.

**Results:**

Beside the altered expression of several miRNAs, our analyses revealed the differential expression of small nucleolar RNAs and misc-RNAs.For the first time, we found that the expression level of miR-26a-5p changed related to INF-β response. MiR-26a-5p expression was significantly higher in IFN-β treated RRMS patients at 3 months treatment, keeping quite stable at 6 months treatments.

**Conclusions:**

Our results might provide insights into the mechanisms of action of IFN-β treatment in MS and provide fundamentals for the development of new biomarkers and/or therapeutic tools.

## Background

Autoimmune diseases as Multiple Sclerosis (MS) are characterized by complex genetic traits and patho-mechanisms that translate into clinical heterogeneity [1,2]. Small non-coding RNAs (sncRNAs) are essential post-transcriptional gene regulation elements that are critical to immune system and neurodegenerative diseases [3-5] by affecting mRNA stability and the expression of multiple genes. Therefore, it is becoming increasingly evident that sncRNA species, as microRNAs, are associated with the development and progression of MS disease [6,7].

The current standard treatment for SM is interferon ß (IFNß) that is less than ideal due to side effects. To improve the efficacy of treatments for MS, it is desirable to find biomarkers that allow early identification of treatment responders and foresee responder status. Thus, better understanding of miRNA and sncRNAs role in MS development and treatment, might contribute to the accumulation of data to understand MS pathogenesis as well as potential approaches for new therapeutic managing.

To shed light into the mechanisms of action of IFN-β treatment in MS, and provide fundamentals for the development of new biomarkers and/or therapeutic tools, we aimed to identify non-coding small RNAs expressed by IFN-β responder treated MS patients. Therefore, we generated small RNA complementary DNA (srcDNA) libraries and sequenced 3000 srcDNA clones. We identified 6 mature miRNAs, 44 C/D box, 2 H/ACA box snorRNAs, 5 antisense fragment and 5 misc-RNAs classes from the peripheral blood mononuclear cell (PBMC) from Relapsing-Remitting MS patients at the baseline and after 3 and 6 months IFN-β treatment. Following, by Real-Time qPCR assay, we assessed, that, among the identified microRNAs, hsa-mir-26a-5p expression was significantly higher in IFN-β treated RRMS patients at 3 months treatment, keeping quite stable at 6 months treatments. We found, for the first time, that the expression of a specific miRNA, hsa-mir-26a-5p, changed during INF-β treatment in responder RRMS patients. Functional annotations of hsa-mir-26a-5p targets revealed that several genes were implicated in Glutamate Receptor Signaling pathway, which is notoriously altered in neurodegenerative diseases as MS.

## Methods

### Ethics statement

We obtained ethics approval for our study from the ethics committee (also known as an Institutional Review Board) at our institution. All the participants had the capacity to consent and we obtained written informed consent from all participants involved in the study.

### Patients with relapsing-remitting MS and RNA isolation

We enrolled 20 patients with a diagnosis of MS according to McDonald criteria [8], all responders to INF-β Standard clinical criteria of response to interferon beta therapy were applied. Patients were considered responders if there was no increase in the EDSS score and no relapses during the follow-up period [9-11]. Additional 10 MS non-responder patients, which were characterized by failure to respond optimally from initiation of therapy as assessed by clinical measures, were recruited too.

Patients (naïve to the therapy) were either treated with interferon-β and blood samples were obtained at a fixed time during the day just before treatment and 3 and 6 months after start of the therapy. Peripheral blood was obtained by venipuncture and immediately processed for isolation of PBMCs. PBMCs were isolated from EDTA blood via ficoll density gradient centrifugation. 1x10^7^ cells were resuspended in QiazolH (Qiagen, Hilden, Germany) and stored at -80°C. Total RNA was extracted using the TRIzol reagent (Invitrogen Carlsbad, CA). RNA samples were quality-checked by identification of 18S rRNA and 28S rRNA peaks via the Agilent 2100 Bioanalyzer platform (Agilent Technologies).

### Small RNA libraries preparation and sequencing

Construction of three small RNA libraries was carried out as previously described [12]. Total RNA (30 μg) was pooled in 3 pools, each comprising RNA from peripheral blood leukocytes of 20 MS patients, at baseline and 3 and 6 months after the start of interferon-β treatment. RNA pools were size fractionated on 15% Tris/Borate/EDTA urea polyacrylamide electrophoresis gel, and the small ncRNA fraction in the size range of 18 to 80 nt was extracted, purified and cloned. Roughly 1000 cloned sncRNA molecules were independently sequenced for each library. Colony PCR was performed using 5′ and 3′ primers, and the clones with PCR products about 110 bp in length were sequenced.

### Biological software analysis

The sequence annotation was based on information from GenBank (http://www.ncbi.nih.gov/Genbank/index.html), a dataset of human tRNA sequences (http://lowelab.ucsc.edu/GtRNAdb/Hsapi/), a dataset of human sn/snoRNA sequences (Small RNA Database, 37 snoRNA-LBME-db at http://www-snorna.biotoul.fr/index.php, NONCODE v138 and the data base from http://research.imb.uq.edu.au/rnadb/), a database of miRNAs (http://microrna.sanger.ac.uk/sequences/index.shtml; release version 7.1).

### Target gene prediction of miRNAs

Target genes for each miRNA were predicted using intersection between at least three of the eleven established target prediction programs compiled by mIRecords, such as TargetScan (http://targetscan.org), PicTar (http://pictar.mdc-berlin.de), miRDB (http://mirdb.org/miRDB/) [13]. To determine genes which were similarly identified by two or more of these algorithmsm, the “microRNA Target Filter” program from IPA (https://analysis.ingenuity.com) was used.

### Real-time qPCR for miRNA quantification

Quantitative real-time RT-PCR was performed using RNA extracted from peripheral blood leukocytes of 20 INFβ-treated MS patients already used for cloning and from 20 additional INFβ-treated MS patients at baseline, 3 and 6 months after the starting of the treatment. Beside, Real-time qPCR for miRNA quantification was also performed using RNA extracted from peripheral blood leukocytes of the 10 INFβ-treated MS non-responder patients at baseline, 3 and 6 months after the starting of the treatment. To quantify miRNA expression levels, microRNA assay-based quantitative RT-PCR (qRT-PCR) was performed for the mature miRNAs, using miScript II RT Kit and miScript SYBR Green PCR kit (Quiagen). Real-time PCR was performed using a 7500 Fast Real-Time PCR system (Applied Biosystems, Foster City, CA, USA. RNU6B was utilized for an endogenous reference to standardize miRNA expression levels.All samples were analyzed in triplicates. All the data were calibrated by the universal reference data. miRNAs expression values were calculated according to the 2^-ΔΔCT^ method. Statistical significant differences were tested by one way analysis of variance (ANOVA), and, when the *F* value was significant, by Student-Newman-Keul’ s test. *P* value less than 0.05 (*) was considered statistically significant.

### Real-time qPCR for mRNA quantification

To confirm the expression patterns of target genes, we performed a quantitative RT-PCR using the comparative C_t_ method. Quantitative real-time RT-PCR was performed using RNA extracted from the 20 INFβ-treated MS patients already analyzed for cloning and from 20 additional INFβ-treated MS patients at baseline, 3 and 6 months after the starting of the treatment.

Transcript levels of the target genes were normalized to β-actin (the internal control) after correcting for differences in amplification efficiencies. RT-PCR reactions (*n* = 3) were performed for each gene of interest using a quantitative real-time Light-Cycler (Roche). All genes investigated have previously been identified and sequences were available in GenBank. Primers for qRT-PCR analysis were designed using the Primer3 program (http://www.bioinformatic.nl/cgi-bin/primer3plus/primer3plus.cgi).

The final PCR reactions contained: 0.4 μM of each primer; 0.25 × SYBR Green (Invitrogen); 4 mM MgCl_2_ and as template 5 μl of cDNA reverse transcribed from a standardized amount of total RNA (0.3 μg). qRT-PCR was performed using Hotstart Taq polymerase (Qiagen) in a final volume of 20 μl. All quantitative reactions were subjected to: 95°C for 15 min followed by 45 cycles at 94°C for 15 s, 59°C 15 s and 72°C 15 s. Melting curve analysis was applied to all reactions to ensure homogeneity of the reaction product. In addition, the amplicon size was checked electrophoretically for each primer set and subsequent sequencing revealed that it corresponded to the human DLG4, HOMER1, GRIN3A, SLC1A1, SLC38A1 transcripts respectively, thus verifying the identity of the genes. Potential contamination was assessed by including non-reverse transcribed total RNA (genomic DNA contamination) and controls without template, observing no products in these reactions.

## Results

### Small non coding RNAs distribution in libraries from various IFN-β treatment stages

**Figure 1:**
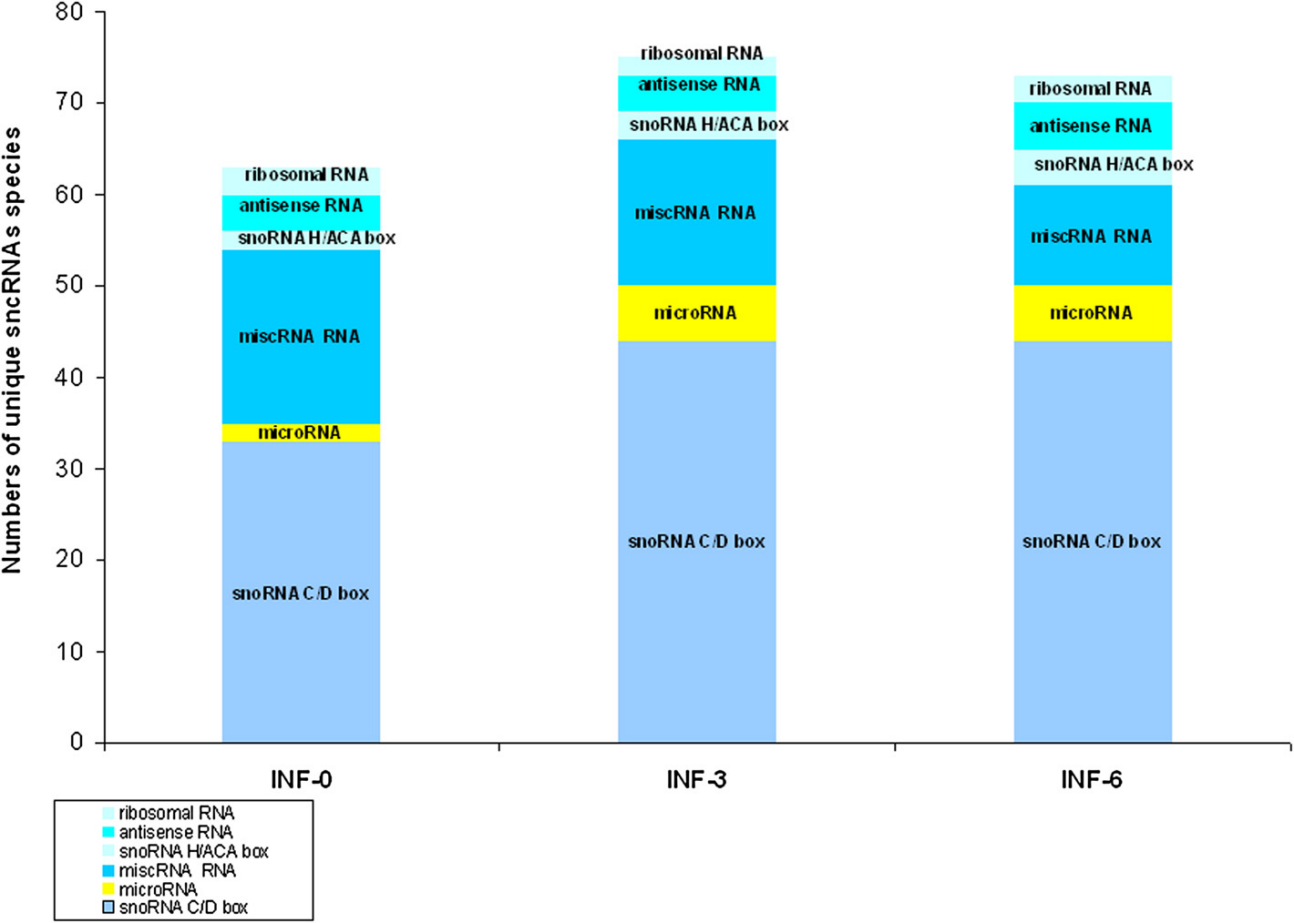
**Sequencing analysis of differentially expressed small sncRNAs.** Sequencing analysis of differentially expressed small sncRNAs in the peripheral blood of Relapsing-Remitting MS patients under IFN-β therapy, at starting, at 3 and 6 months treatment (Library 1, 2, 3 respectively) (a) Number of sncRNA species per sample pool.

With the aim of characterizing the sncRNA signature from the peripheral blood of Relapsing-Remitting MS patients under IFN-β therapy, a set of 20 cases (Table 1), were evaluated by sncRNA cloning at starting, at 3 and 6 months treatment (IFN-0, IFN-3, IFN-6 libraries respectively). About 1000 cloned sncRNA molecules were independently sequenced for each library. This approach allowed us to obtain a profile of sncRNAs expressed in these cell types at different treatment stages (Figure 1).

**Table 1:** General characteristics of the study subjects

**Number of samples**		**Age range (years) +/-SD**	**Disease duration range (years)+/-SD**	**EDSS range +/-SD**
Total	40	17-41+/-3	2-6+/-2	1-4.7+/-2
Male	18	18.3-42+/- 2	2-5+/-2	1-4.5+/-2
Female	22	19-39+/-3	1.8-6+/-3	1-5.0 +/-3

Identified loci were further classified as known miRNA, snoRNA, small ribosomal RNA, tRNA, antisense fragment ncRNAs. We were able to isolate 6 mature miRNA species deriving from known human stem-loop sequences (Table 2). SnoRNAs were the most abundant ncRNA species detected, comprising approximately 61% of the total RNA pool (Figure 1, Table 2). A search with the mapped sequences from the sample libraries against known snoRNAs identified 44 unique snoRNA loci encoding C/D-box and 2 H/ACA box snoRNAs (Table 2). The diversity of detected snoRNA species was increased in the IFN-0 library, H/ACA box snoRNAs were detected in IFN-3 and IFN-6 libraries (Figure 1). Moreover, five different unique antisense RNA fragments and miscRNA classes, respectively were isolated from these libraries.

**Table 2:** Small non-coding RNA classes identified by cloning

**SNORD C/D**	**SNORD H/ACA**	**Antisense RNA**	**miRNAs**	**misc-RNA**
C/D box 30	H/ACA box 16A	C17orf76 antisense RNA 1 transcript variant 5	miR-26a-5p	LOC100130249
C/D box 49A	H/ACA box 16B	C17orf76 antisense RNA 1 transcript variant 6	miR-3676	LOC100653240
C/D box 80		C17orf76 antisense RNA 1 transcript variant 7	miR-326	LOC100652939
C/D box 77		C17orf76 antisense RNA 1 transcript variant 13	miR-155	LOC100506834
C/D box 100		C1orf213 transcript variant 1 non-coding RNA	miR-18b	LOC100506679
C/D box 49B			miR-599	
C/D box 2				
C/D box 50A				
C/D box 81				
C/D box 69				
C/D box 95				
C/D box 60				
C/D box 12B				
C/D box 68				
C/D box 27				
C/D box 59B				
C/D box 105B				
C/D box 73A				
C/D box 41				
C/D box 1C				
C/D box 47				
C/D box 82				
C/D box 12C				
C/D box 52				
C/D box 12				
C/D box 26				
C/D box 101				
C/D box 102				
C/D box 68A				
C/D box 87				
C/D box 88C				
C/D box 88B				
C/D box 88A				
C/D box 57				
C/D box 85				
C/D box 59A				
C/D box 127				
C/D box 4B				
C/D box 74				
C/D box 29				
C/D box 70				
C/D box 111B				
C/D box 105				
C/D box 45C				

### miRNAs identified in different stages of IFN-β treatment

The overall expression of miRNA species was relatively increased after 3 months therapy as demonstrated by the noticeably higher total sequence count of miRNAs in IFN-3 and IFN-6 compared to INF-0 library. We were able to identify 6 mature miRNA species, miR-326, miR-155, miR-3676, miR-18b, miR-599 and miR-26a-5p deriving from known human stem-loop sequences (miRBase 12.0).

### The expression of miR-26a-5p is increased in the blood of IFN-β treated SM patients

**Figure 2:**
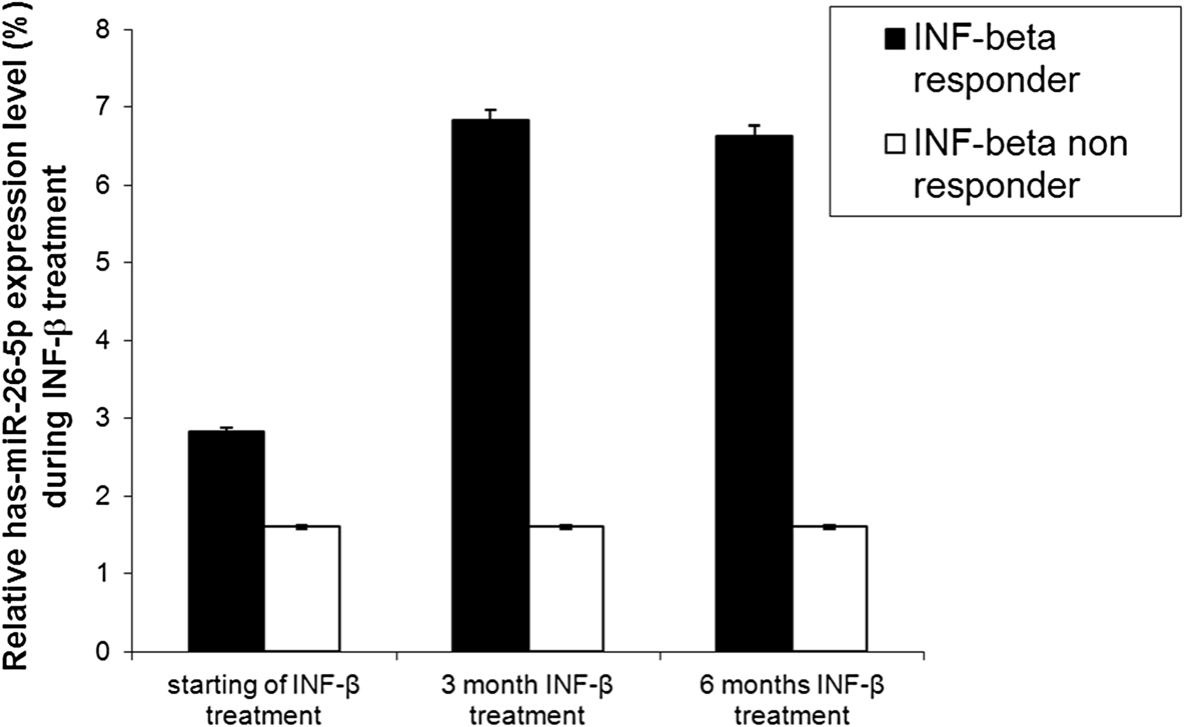
**MiR-26a-5p expression levels in INF-β treated MS patient leukocytes.** The expression of mir-26a-5p was studied in the peripheral blood from 40 IFN-β treated responder RRMS patients and from 10 IFN-β treated non-responder patients, at starting, at 3 and 6 months IFN-β treatment by microRNA assay-based quantitative RT-PCR following the 2^-ΔΔCT^ method. RNU6B was utilized for an endogenous reference to standardize microRNA expression levels. The results were expressed as relative expression levels after calibration with the universal reference data. P < 0.05.

To validate the results obtained by cloning and to acquire a more quantitative profile of the expression, the six miRNAs expression was measured in 20 patients already analyzed by cloning and in 20 additional patients by qRT-PCR, during baseline and 3 and 6 months IFN-β. treatment.Among these, only hsa-miR-26a-5p, which has been shown to be mainly expressed in neural tissue [14], showed the most significant expression change in IFN-β treated RRMS patients, at different therapy stages.MiR-26a-5p expression was significantly higher in IFN-β treated responder RRMS patients at 3 months treatment compared to the baseline, keeping quite stable at 6 months treatments (Figure 2). MiR-26a-5p expression did not show any significant expression change in a group of IFN-β treated non-responder MS patient (Figure 2).

### Computational predictions of the putative targets of miR-26a-5p

**Figure 3:**
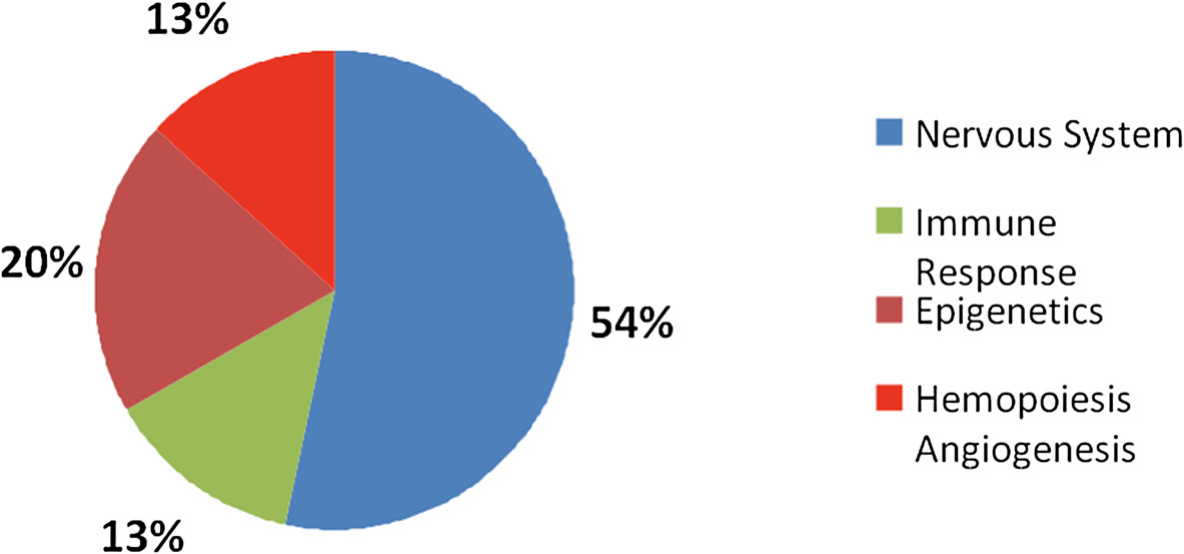
**Gene networks regulated by miR-26a-5p.** Predicted miR-26a-5p target genes identified by TargetScan, PicTar , Miranda and miRBase Target database search.

The overlap between putative targets of miRNAs and the expression of mRNAs provides information on the biological functions and networks of genes regulated by specific miRNAs. Therefore, we explored putative miR-26a-5p target genes by searching them on three distinct web-accessible miRNA target databases, including TargetScan, PicTar, and miRDB [13]. Ingenuity Pathway Analysis (IPA) software identified biological pathways associated with predicted miR-26a-5p targets. When we looked for the most significant associations of target genes with different categories of biological functions, we found genes shared among the three databases, mainly involved in Nervous System (54%), Immune Response (13%), Epigenetics (20%), Hemopoiesis and Angiogenesis (13%) (Figure 3). Interestingly, a subset of mir-26a-5p potential genes target, selected by “MicroRNA Target Filter” program from IPA, were implicated in Glutamate Receptor Signaling metabolism (Table 3), which is altered in brains of multiple sclerosis patients [15]. Indeed, , the following hypothetical candidate genes of miR26a-5p have been identified as target genes, DLG4, HOMER1, GRIN3A SLC1A1, SLC38A1 (Table 3).(DLG4), a key player in neuronal and glutamate signaling [16] HOMER1 (homer homolog 1) and GRIN3A (glutamate receptor ionotropic N-methyl-d-aspartate 3A), are all related to glutamate signaling [17].

**Table 3:** Predicted miR-26a-5p target genes, involved in glutamate receptor signaling pathway, identified by TargetScan, MiRDB and PicTar target database search and selected by “MicroRNA Target Filter” program from IPA

**Gene ID**	**Gene symbol**	**Gene name**	**Target scan score**	**Pictar score**	**MiRDB score**
NM_001128827	DLG4	discs, large homo log 4 (Drosophila)	-.29		82
NM_004272	HOMER1	homer homolog 1 (Drosophila)	-0.13		
NM_133445	GRIN3A	glutamate receptor, ionotropic, N-methyl-D-aspartate 3A	-0.54		
NM_004170	SLC1A1	solute carrier family 1 (neuronal/epithelial high affinity glutama te transporter, system Xag), member 1	-0.82	1.57	
NM_001077484	SLC38A1	solute carrier family 38, member	-0.07		

### Differential expression of miR-26a-5p gene targets in the blood of IFN-β treated SM patients

**Figure 4:**
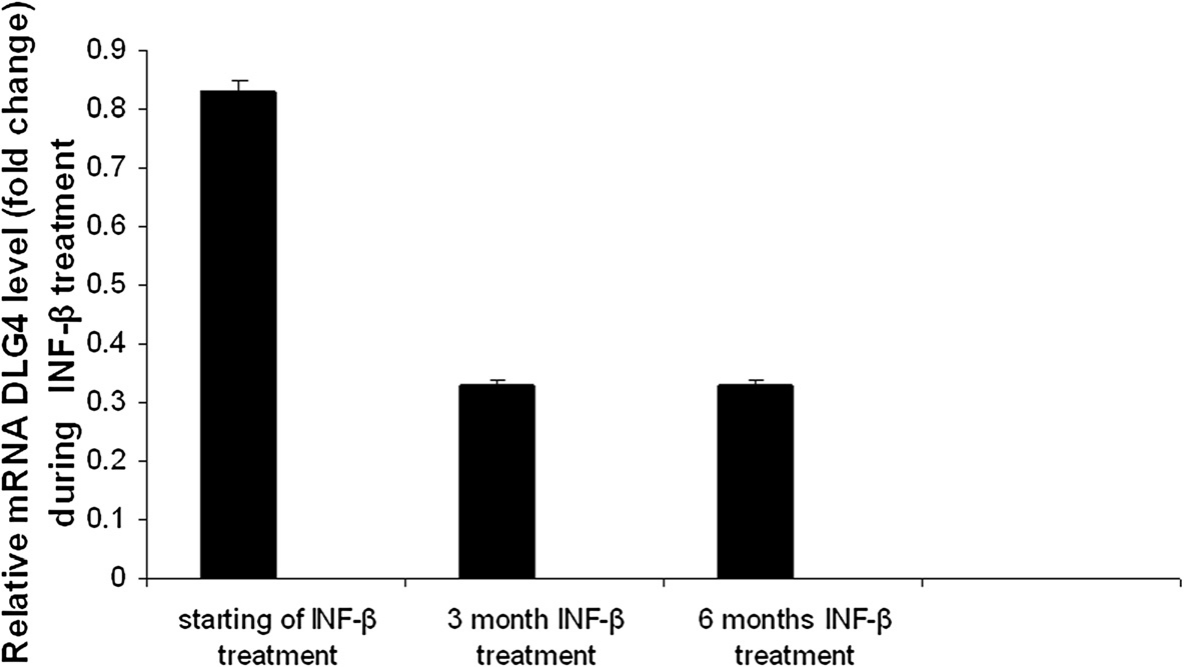
**DLG4 expression levels in INF-β treated MS patient leukocytes.** Significant up-regulation of the expression of putative miR-26a-5p targets, DLG4, was evaluated by qPCR in INF-b treated MS patients leukocytes. P < 0.05.

**Figure 5:**
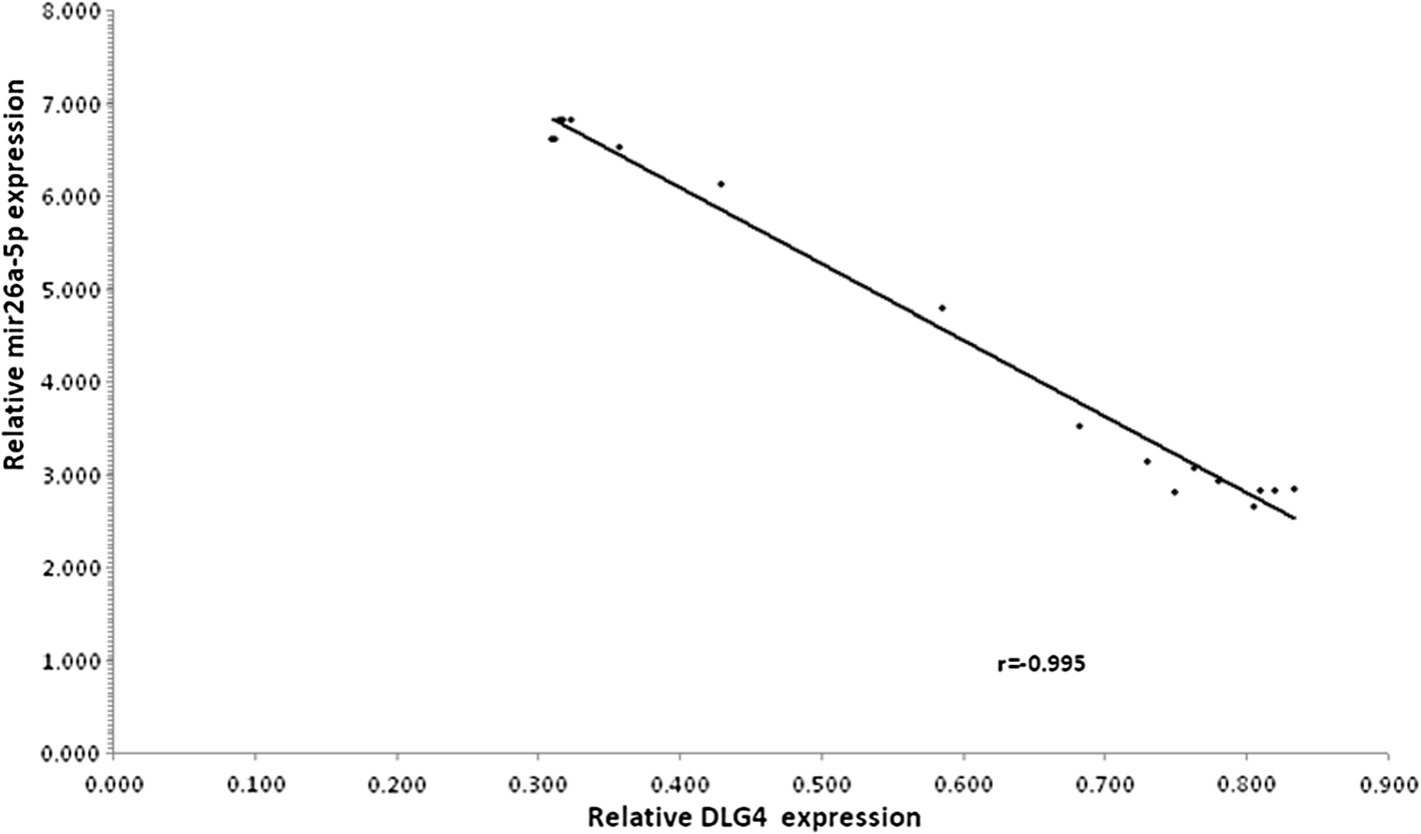
**Correlation of miR-26a-5p and DLG4 expression level in MS patients leukocytes after 6 months IFN-β treatment.** The expression level of miR-226a-5p and DLG4 was determined by quantitative Real Time RT-PCR. A significant correlation between miR-26a-5p and DLG4 expression levels was observed (Pearson’s correlation coefficient = -0.995, P = 0.003).

The expression of miR-26a-5p gene targets, representing key role in Glutamate Receptor Signaling biological pathway selected by “MicroRNA Target Filter” program from IPA (Table 3), was evaluated by qPCR. Among the subset of five gene target, DLG4 showed a significant expression change in blood samples from IFN-β treated RRMS patients, at different treatment stages. We were able to detect 2.5 fold decreases in DLG4 expression in the blood from 3 months IFN-β treated SM patients, keeping quite stable at 6 months (Figure 4). As shown in Figure 5, after 6 months IFN-β treatment, DLG4 expression showed a significant negative correlation to miR-26a-5p expression (Pearson’s correlation r = - 0.995 (P = 0.003). Since DLG4 contains a 8-nt miR-26a-5p binding site in its 3′ UTR, these results might suggest that miR-26a-5p up-regulation could have a significant impact on the regulation of DLG4 gene expression in INF- β treated responder subjects.

## Discussion

At present, consistent data regarding alterations in the expression of miRNAs in MS are reported [18-20]; however, studies addressing the genome-wide expression profiling of other small sncRNA in this disease are lacking. Moreover, little is known about small regulatory RNAs and how they contribute to pathogenesis, progression and transcriptome changes in MS and to the mechanisms of action of IFN-β treatment in MS patients. This information can be utilized as a novel biomarker to predict identification of INF-β responders during the therapy as well to be applied to develop novel therapy for MS patients.

Here, we detected a pronounced increase in the three libraries for several snoRNAs, providing further support for high metabolic activity and elevated protein-synthesis rate. Interestingly, in addition to their function in ribosomal maturation, snoRNA species or their derivates have been implicated in processes associated to alternative splicing events [21] and genomic imprinting [22]. Recent findings have demonstrated that snoRNAs can also function as miRNAs [23]. The HBII-52 C/D box snoRNA associated with Prader-Willi syndrome is one important example [24]. Additional research is needed to investigate whether this unconventional function of snoRNAs is a large spread mechanism of gene regulation and whether this keeps true for snoRNAs identified in this research.

Five different antisense RNA fragment and 5 miscRNA classes were isolated in these libraries. Lately it has been shown that antisense RNA transcripts are associated not only to imprinting and X inactivaction, but can occur at non-imprinted autosomal loci. This mechanism can have a role in the regulation of gene expression in normal conditions as well as in inherited disorders [25]. Our screening revealed that blood miRNA expression can be associated with patient’s INF-β response. Not all MS patients respond to interferon beta therapy, and to date, there is a lack of markers that can correlate with responsiveness to interferon-β therapy in MS. In a previous study, the expression of five selected miRNAs in a group of glatiramer acetate and interferon-beta treated MS patients, from peripheral blood mononuclear cell, has been evaluated to detect a potential impact of immuno-modulatory therapy on deregulated miRNAs [26]: however, miRNAs expression did not differ between treatment-naıve and IFN-beta treated RRMS patients. In our research, mir-26a-5p, showed a significant change in the level of expression, based on the INF-βresponse, at the different stages of treatment in INF-β treated responder RRMS (Figure 2). Studies have demonstrated that glutamatergic regulation can take place in MS symptoms and treatment [27]. Under pathological conditions an excess of glutamate in the synaptic space can trigger a toxic cascade leading to cell death. This neuro-excitotoxicity cascade might have a key role linking the presence of inflammation to axonal injury in multiple sclerosis [28].

Interferon-β (IFN)-β reduces symptoms of the relapsing–remitting form of MS. Beside the effects on immuno-modulatory system and metalloproteinase 9 [29], IFN-β has been shown to be neuroprotective against the toxicity induced by activated microglia in cortical neurons and microglia co-cultures, suppressing the production of glutamate and superoxide by activated microglia [30] Considering that the expression level mir-26a-5p changed, these variations can have a significant impact on a patient’s INF - β-response and glutamate activity. The expression levels of miR-26a-5p in the response to treatment were consistently and significantly higher in IFN-β treated RRMS patients at 3 months treatment, keeping stable at 6 months treatments in all patients. Beside, qPCR results have demonstrated that DLG4 expression decreased after 3 months treatment, showing a significant inverse correlation to mir-26a-5p expression. The DLG4 transcript of 4.2 kb is most abundant in brain, but also expressed in leukocytes [16]. These results are interesting, especially if we consider that post-synaptic density protein 95 (DLG4), a key player in neuronal signaling, has been identified as a putative regulators connecting 64% of the identified proteins in rats induced acute experimental autoimmune encephalomyelitis (EAE), a well characterized disease model of MS [31]. We hypothesized, therefore, that mir-26a-5p might down-regulate the expression level of glutamate-signaling related genes in INFβ- treated MS patients. However, the underlying molecular mechanism warrants further research also in neuronal cells and/or animal model.

## Conclusions

In conclusion, our research revealed that a blood miRNA expression can be associated with a patient’s INF response. Since information on miRNA is regularly being updated, we are planning in the future to perform more analysis using a larger number and different kind of sample as neuronal tissue. As result, miRNA profiling can be useful as a biological marker for predicting the identification of INF-β responders during the therapy, thereby reducing ineffective treatments. Beside, miRNA expression profile can facilitate the accumulation of data to develop novel therapeutic targets for MS [32].

## Competing interests

The authors declare that they have no competing interests.

## Authors’ contributions

BDF, PM, and GO participated in the design of the study and coordination. BDF, EB, MB, RP prepared the samples and performed the assays. AS, GV, VB carried out the statistical analyses included the manuscript. BDF drafted the manuscript. All authors read and approved the final manuscript.

## Pre-publication history

The pre-publication history for this paper can be accessed here:

http://www.biomedcentral.com/1755-8794/7/26/prepub
